# Novel SNPs and InDels discovered in two promoter regions of porcine pregnancy-associated glycoprotein 2-like subfamily (*pPAG2-Ls*) in crossbreed pigs

**DOI:** 10.1007/s10142-016-0522-z

**Published:** 2016-10-05

**Authors:** Martyna Bieniek-Kobuszewska, Grzegorz Panasiewicz, Aleksandra Lipka, Marta Majewska, Bozena Szafranska

**Affiliations:** 1 Department of Animal Physiology, Faculty of Biology and Biotechnology, University of Warmia and Mazury in Olsztyn, Oczapowskiego Str 1A, 10-719 Olsztyn-Kortowo, Poland; 2 Department of Dermatology, Sexually Transmitted Diseases and Clinical Immunology, Faculty of Medical Sciences, University of Warmia & Mazury in Olsztyn, Wojska Polskiego Str 30, 10-229 Olsztyn, Poland; 3 Department of Human Physiology, Faculty of Medical Sciences, University of Warmia & Mazury in Olsztyn, Warszawska Str 30, 10-082 Olsztyn, Poland

**Keywords:** PAG, Polymorphism, Promoter, SNP, TBSF

## Abstract

This is a pioneer study of single nucleotide polymorphisms (SNPs) within the entire promoter region (1204 bp) of the dominant *pPAG2-L* subfamily in the pig. The *pPAG2-L* subfamily was sequenced/examined using genomic deoxyribonucleic acid (gDNA) templates of crossbreed pigs (Landrace x Large White), and compared to two bacterial artificial chromosome (BAC) clones containing gDNA of the Duroc breed (as the positive controls). Our analysis of the *pPAG2-L* promoter identified 31 SNPs and one InDel mutation in crossbreed pigs. Among 42 SNPs identified in two BAC clones, 24 SNPs had not been previously detected in crossbreed pigs. The sequence alignment of *pPAG2-L* promoter, performed with Lasagne-Search 2.0, Cluster Bluster and MatInspector software, revealed a total of 28 transcription factor binding sites (TFBS) and 10 TFBS (AP-1, CCAAT, CHOP:C, FOXP1, LSF, MRF-2, Myc, NF1, NF-Y, TGIF) within SNPs in the core sequences. It was noted that TFBS (NF1) was found to be unique to the *pPAG2* promoter sequence containing SNPs: g.-1100G>A(R), g.-1101T>C(Y), represented by GA and TC genotypes (*p*_*x*_ = 0.12). Our broad-based novel database thus provides an SNP *PAG2-L* pattern for modern genotyping of female and male progenitors. This is required for further studies of various potential correlations between guiding SNP genotypes of the *pPAG2-L* subfamily in the sows of many breeds, in which the most economically important reproductive traits are properly documented on each farm.

## Introduction

Multiple pregnancy-associated glycoprotein family (PAGs) belongs to placental aspartic peptidases that have been identified mainly by complementary DNA (cDNA) cloning in some eutherian taxa only (see Xie et al. 1997; Szafranska et al. 2006; Wallace et al. 2015). In the domestic pig (Szafranska et al. 1995; Panasiewicz et al. 2004), among eight trophoblastic cloned and identified cDNAs (GenBank: L34360–1, AF315377, AF272734, AY188554, AF272735, AY373029 and AY775784), five are classified as catalytically active porcine (p) *PAG2-L* subfamily (*pPAG2, -4, -6, -8* and *-10*) or potentially inactive *pPAG1-L* subfamily (*pPAG1, -3* and *-5*). In the pig (Cetartiodactyla), both subfamilies have been identified in trophoblastic cells (TR) during the peri-implantation period and in trophectoderm cells (TRD – chorionic epithelium) after post-placental development (Szafranska and Panasiewicz 2002; Szafranska et al. 2006).

The *PAG* family encodes multiple chorionic polypeptide precursors that start expression during peri-implantation, the period of the highest embryonic mortality in many eutherians (Xie et al. 1997; Szafranska et al. 2006; Wallace et al. 2015). All identified *PAG* cDNAs permitted genomic DNA (gDNA) cloning and initial exon-intron structure organization discoveries of nine exons and eight introns in the cattle and the pig (Xie et al. 1995; Szafranska et al. 2001). To date, 11 promoter sequences of the *PAG* family have been identified: bovine* – bPAG1* (Xie et al. 1995), *bPAG2, 3, 5, 8, 11, 12, 15,* and *18* (Telugu et al. 2009), porcine* – pPAG2* (Szafranska et al. 2001), and equine* – ePAG* (Green et al. 1999). In the pig, the single nucleotide polymorphisms (SNPs) within the promoter of the *pPAG2* and either *pPAG2-L* or *pPAG1-L* subfamilies have not yet been identified.

Despite all available information concerning the promoter sequences of the *PAG* family, there is no information on polymorphism or the potential influence of SNPs on the regulation of transcription. The present study was conducted to identify polymorphisms in the promoter region of the *pPAG2-L* subfamily and to determine the genotype frequency in crossbreed and Duroc pigs (as controls). Such examined SNPs in the crossbreed pigs will provide the main pattern for the genotyping of multiparous sows of many breeds, in which reproductive traits are known, which is economically important in the livestock industry.

## Materials and methods

### Animals and genomic DNA templates

Blood samples (40 ml in to the tubes with K_2_EDTA) were collected from female (*n* = 6) and male (*n* = 11) crossbreed pigs (Landrace x Large White) slaughtered under commercial conditions. All gDNA templates were isolated by the Sherlock AX procedure (A&A Biotechnology, Poland) and used for various amplicon productions by PCR with primer sets (F:GGCTTATCTGTCCCCACTGG and R:AGTAAGACACAGGCAGTC or F:GACTGCCTGTGTCTTACT and R:GACTGTCAGGAATGATGGCA), specific for the *pPAG2* promoter (Acc. No. U39198/GenBank; Szafranska et al. 2001). The PCR conditions were initial denaturation at 94 °C/3 min; then 40 cycles 94 °C/1 min, 62 °C/1 min, 72 °C/2 min, and the final extension at 72 °C/7 min. All PCR mixes (10 μl) contained 0.42 μl dNTP; 0.4 μl 25 mM MgCl_2_; 2 μl 10× buffer; 0.4 μl JumpStart™Taq DNA Polymerase (Sigma-Aldrich, USA); 0.7 μl of each primer (100 ng/μl); and various gDNA templates of the crossbreed pigs (200 ng), parallel to gDNA bacterial artificial chromosome (BAC) clones (5 ng) specific only for the Duroc breed (CH242-60C13 and CH242-294016; BACPAC Resources, CHORI, Children’s Hospital Oakland Research Institute, USA) – as the positive controls.

### Preparation of the positive controls (gDNA) – BAC clones

Two commercial BAC clones (CH242-60C13 and CH242-294016) were propagated in transformed *Escherichia coli* strain DH10B bacteria containing gDNA inserts (279 330 and 97 794 bp, respectively) in the pTARBAC1.3 vectors (13 462 bp). The DH10B bacteria were grown in NZY Broth medium supplemented with chloramphenicol (12.5 mg/ml medium), and plasmid DNA (plDNA) was harvested by standard alkaline lysis. Isolated plDNAs were spectrophotometrically assessed and used for PCR amplifications, as described above.

### Sequencing and SNP identification within *pPAG2-L* promoters

All *pPAG2-L* amplicons were separated in 1 % agarose gels, purified according GenElute™ Gel Extraction Kit procedure (Sigma-Aldrich, USA) and sequenced in both sense and anti-sense directions by 3130 Genetic Analyzer using the BigDye^®^ Terminator v.3.1 Cycle sequencing procedure (Applied Biosystems, USA). The obtained chromatograms were initially examined by Sequencing Analysis Software (Applied Biosystems, USA), and all sequences were verified by FinchTV (Geospiza, Inc., USA) and aligned by DNASIS v.3.0 (Hitachi Software Engineering Co. Ltd., Japan) and the National Center for Biotechnology Information Basic Local Alignment Search Tool (NCBI BLASTn) using discontinuous Megablast or Blastn in the GenBank. All identified SNPs were named according to the International Union of Pure and Applied Chemistry (IUPAC) codes.

### Computer analysis

*In silico* analysis of the *pPAG2-L* promoter sequences for a presence of putative TFBS was performed by Cluster Bluster, MatInspectior™ and Lasagna-Search 2.0 with TRANSFAC matrices. The analyses were carried out for all possible TFBS according to the individual settings of each software: Cluster Bluster (Gap Parameter 35; Cluster Score Treshold 2; Motif Score Treshold 2; Residue Abundance Range 100, Pseudocount 0.375), MatchInspector (minimize false positives), Genomatix RegionMiner tool for overrepresentation of TFBSs (Genomatix Software GmbH), and Lasagna-Search 2.0 (*p* ≤ 0.001).

## Results

### Identifications of sequences and novel SNPs within the *pPAG2-L* promoter in the crossbreeds

**Fig. 1:**
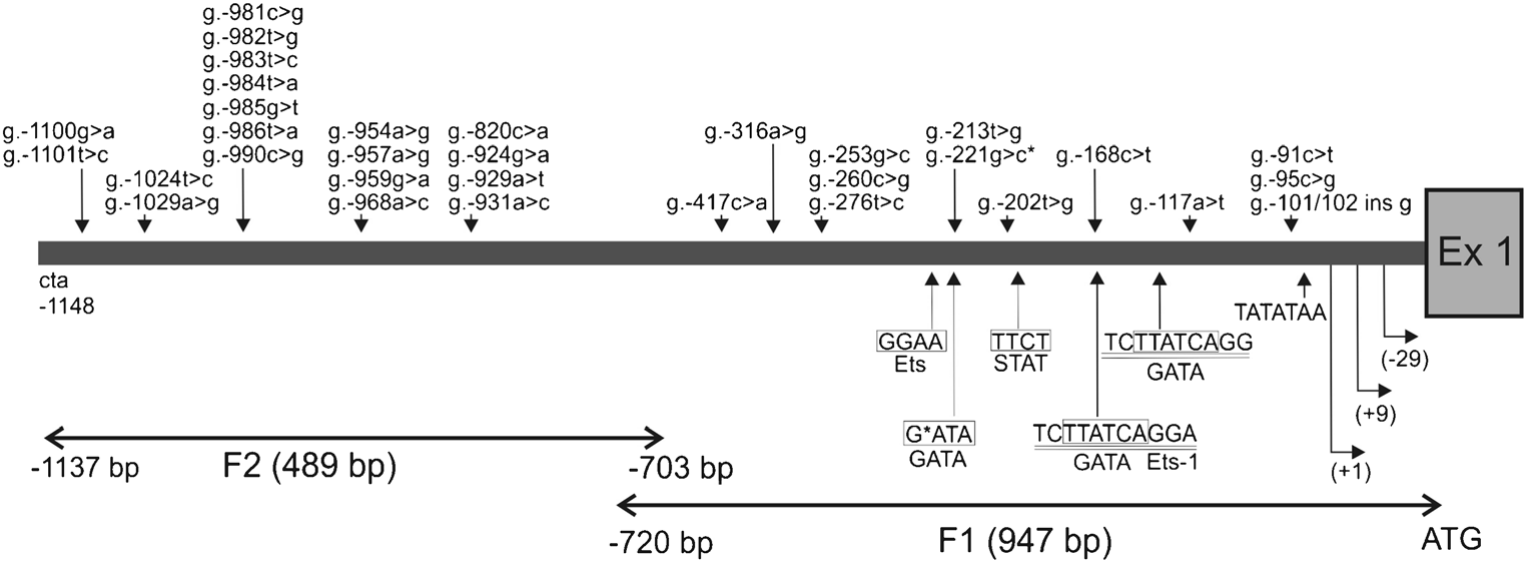
Schematic localization of the SNPs in the promoter sequence (1204 bp upstream from ATG) of the *pPAG2-L* subfamily examined in the crossbreed pigs. This figure includes the transcription start site (+1, +9, −29); potential binding sites for transcription factors - Ets, GATA, STAT (*boxed*); TATA-box (TATATAA); unique tandem repeats (*double underlined*); the occurrence of SNP (*p*.−168 g > c*) in the coding sequence for GATA

In total, 31 novel SNPs located from *g.-91C>T(Y)* to *g.-1101T>C(Y)* plus one InDel mutation (*g.-100/101InsG*) upstream ATG were identified in the promoter region of the *pPAG2-L* subfamily (Fig. 1, Table 1). This provides a novel major pattern of the largest genetic variation of the porcine genome due to various crossbreeds. All SNPs were submitted to the dbSNP/NCBI database and analyzed according to the 1204 bp of the *pPAG2* promoter (Acc. No. U39198; GenBank). The SNPs were identified within two promoter fragments, including F1) 947 bp proximal regulatory region (-720 bp upstream ATG) and F2) 489 bp flanking distal region (from – 703 to – 1137 bp upstream ATG). Among 32 SNPs/InDel, 13 SNPs were identified in the F1 and 19 SNPs in the F2. Within the F1, one insertion (*g.-100/101InsG*) and 12 SNPs (4 transitions – TRNs and 8 transversions – TRVs) were identified, while among 19 SNPs in the F2, we detected 9 TRNs and 10 TRVs.

**Table 1:** Genotype frequencies (*p*_*x*_) of the identified SNPs in the promoter of the *pPAG2-L* gene subfamily in crossbreed pigs

SNP locus (IUPAC code)^a^		SNP locus acc. DNASIS^b^	Genotypes and frequencies (*p*_*x*_)					
			Genotype	*p*_*x*_	Genotype	*p*_*x*_	Genotype	*p*_*x*_
F1 region								
1)	*g.-91C>T (Y)*	*g.1111C>T*	CC	0.45	CT	0.55	TT	0.0
2)	*g.-95C>G (S)*	*g.1107C>G*	CC	0.45	CG	0.55	GG	0.0
3)	*g.-101/102->INSG*	*g.1100/1101->InsG*	GG	1.0	–/–	0.0	–/–	0.0
4)	*g.-117A>T (W)*	*g.1085A>T*	AA	0.57	AT	0.43	TT	0.0
5)	*g.-168C>T (Y)*	*g.1034C>T*	CC	0	CT	1.0	TT	0.0
6)	*g.-202T>G (K)*	*g.1000T>G*	TT	0.56	TG	0.44	GG	0.0
7)	*g.-213T>G (K)*	*g.989T>G*	TT	0.14	GT	0.86	GG	0.0
8)	*g.-221G>C (S)*	*g.981G>C*	GG	0.14	CG	0.86	CC	0.0
9)	*g.-253G>C (S)*	*g.949G>C*	GG	0.17	CG	0.83	CC	0.0
10)	*g.-260C>G (S)*	*g.942C>G*	CC	0.0	CG	1.0	GG	0.0
11)	*g.-276T>C (Y)*	*g.926T>C*	TT	0.0	CT	1.0	CC	0.0
12)	*g.-316A>G (R)*	*g.886A>G*	AA	0.2	AG	0.2	GG	0.6
13)	*g.-417C>A (M)*	*g.785C>A*	CC	0.0	AC	1.0	AA	0.0
F2 region								
14)	*g.-820C>A (M)*	*g.382C>A*	CC	0.2	AC	0.4	AA	0.4
15)	*g.-924G>A (R)*	*g.278G>A*	GG	0.71	AG	0.29	AA	0.0
16)	*g.-929A>T (W)*	*g.273A>T*	AA	0.71	AT	0.29	TT	0.0
17)	*g.-931A>C (M)*	*g.271A>C*	AA	0.71	AC	0.29	CC	0.0
18)	*g.-954A>G (R)*	*g.248A>G*	AA	0.0	AG	1.0	GG	0.0
19)	*g.-957A>G (R)*	*g.245A>G*	AA	0.0	AG	1.0	GG	0.0
20)	*g.-959G>A (R)*	*g.243G>A*	GG	0.0	AG	1.0	AA	0.0
21)	*g.-968A>C (M)*	*g.234A>C*	AA	0.12	AC	0.82	CC	0.06
22)	*g.-981C>G (S)*	*g.221C>G*	CC	0.76	CG	0.24	GG	0.0
23)	*g.-982T>G (K)*	*g.220T>G*	TT	0.76	GT	0.24	GG	0.0
24)	*g.-983T>C (Y)*	*g.219T>C*	TT	0.76	CT	0.24	CC	0.0
25)	*g.-984T>A (W)*	*g.218T>A*	TT	0.76	AT	0.24	AA	0.0
26)	*g.-985G>T (K)*	*g.217G>T*	GG	0.76	GT	0.24	TT	0.0
27)	*g.-986T>A (W)*	*g.216T>A*	TT	0.0	AT	1.0	AA	0.0
28)	*g.-990C>G (S)*	*g.212C>G*	CC	0.82	CG	0.18	GG	0.0
29)	*g.-1024T>C (Y)*	*g.178T>C*	TT	0.82	CT	0.18	CC	0.0
30)	*g.-1029A>G (R)*	*g.173A>G*	AA	0.82	AG	0.18	GG	0.0
31)	*g.-1100G>A (R)*	*g.102G>A*	GG	0.88	AG	0.12	GG	0.0
32)	*g.-1101T>C (Y)*	*g.101T>C*	TT	0.88	CT	0.12	CC	0.0

^a^Numbering of SNPs submitted in dbSNP/NCBI database

^b^Numbering according to promoter sequence U39198, GenBank (Szafranska et al. 2001)

Genotype frequency varied in homo- and heterozygotes in a range *p*_*x*_ = 0.55–1. Among 31 SNPs and one InDel, 18 SNPs dominated in homozygotes (*p*_*x*_ = 0.56–0.88), whereas 14 SNPs existed in heterozygotes (*p*_*x*_ = 0.55–1). Only one SNP (*g.-221G > C*) localized in the F1, dominated in heterozygotes (*p*_*x*_ = 0.86), changing the sequence of GATA, while other neighboring SNPs did not affect TFBS – GATA. Most of the 19 SNPs identified in the F2 were closely localized in three major groups: (1) 820–931, (2) 954–968, and (3) 981–990 bp, while 4 other SNPs existed in tandems until −1101 bp upstream ATG. Interestingly, among the 32 identified SNPs/InDel, only three identified SNPs in homozygotes (Table 1): 12) *g.-316a>g* (GG); 14) *g.-820C>A* (AA); and 21) *g.-968A>C* (CC) had genotypes existing with frequencies (*p*_*x*_) ranging from 0.06 to 0.6.

**Fig. 2:**
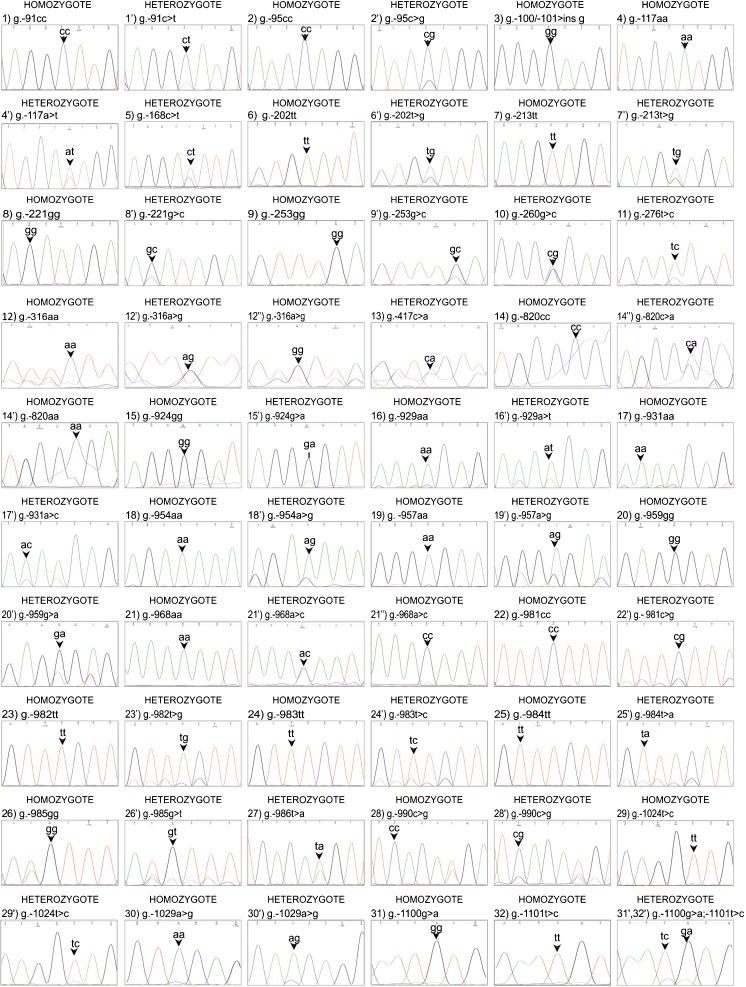
Chromatograms of identified SNPs (*arrows*) in homozygotes and heterozygotes within the promoter sequence region (from g.-91C>T to g.-1029A>G) of the *pPAG2-L* subfamily in crossbreed pigs. [For an interpretation of the references to color in this figure legend, the reader is referred to the web version of this article]

All original chromatograms of 31 SNPs and one InDel identified within both F1 and F2 regions of the *pPAG2-L* promoter (Table 1), including the monoallelic (homozygotes) and biallelic (heterozygotes) visualized by the Finch TV (Fig. 2), revealed sequence differences compared to the only available consensus sequence of the *pPAG2* promoter for various porcine breeds (U39198; Szafranska et al. 2001). In addition, we identified that in the BAC clones (CH242-60C13 and CH242-294016) used as the only available commercial control sequences (for *pPAG3* and *pPAG6*), a surprisingly large diversity was identified for various members of the entire *pPAG* family, including the *pPAG1-L* and *pPAG2-L* subfamilies.

### Identification of sequences and SNPs within the *pPAG2-L* promoter in control BAC clones

Sequencing of the commercial BAC clones (CH242-60C13 and CH242-294016), used as the major positive controls containing only gDNA specific for the Duroc breed, revealed very high sequence diversity, including the presently identified 36 SNPs and 6 InDels (Table 2). Conversely, our parallel broad-based *in silico* analysis of both BAC clone sequences revealed very huge multiplicity and diversity of the entire *pPAG2-Ls* and/or additionally numerous and various fragments (Panasiewicz et al. unpublished data).

**Table 2:** SNP identification in promoter sequence of the *pPAG2-L* gene subfamily within BAC clones: CH242-60C13 and CH242-294016

SNP localization in 1204-bp promoter				BAC clones (Duroc)				Crossbreeds
SNP locus (IUPAC code)^b^		SNP locus acc. DNASIS^c^	*pPAG2*	CH242-60C13^d^		CH242-294016^e^		
				Amplicon 1	Amplicon 2	Amplicon 3	Amplicon 4	
F1 region								
1)	*g.-101 -102Ins* *G*	*g.1100_1101InsG*	*DelG*	GG	–/–	GG	–/–	GG
2)	*g.-168C* *≥* *T(Y)*	*g.1033C>T*	*CC*	TT	–/–	TT	–/–	CC
3)	*g.-316A* *≥* *G(R)*	*g.886 A>G*	*AA*	AG	–/–	AG	–/–	*GG/AG*
4)	*g.-417C* *≥* *A(M)*	*g.785C>A*	*CC*	AC	–/–	AC	–/–	*AA*
F2 region								
*5)*	*g.-740G>T(K)* ^a^	*g.462G>T*	*GG*	GG	TT	GG	TT	GG
*6)*	*g.-810A>G(R)* ^a^	*g.392A>G*	*AA*	AA	GG	AA	GG	AA
7)	*g.-820C* *≥* *A(M)*	*g.382C>A*	**CC**	AC	CC	AC	CC	*AA/CC*
*8)*	*g.-826G>A(R)* ^a^	*g.376G>A*	*GG*	GG	AA	GG	AA	GG
*9)*	*g.-827G>C(S)* ^a^	*g.375G>C*	*GG*	GG	CC	GG	CC	GG
*10)*	*g.-828G>A(R)* ^a^	*g.374G>A*	*GG*	GG	AA	GG	AA	GG
*11)*	*g.-836C>A(M)* ^a^	*g.366C>A*	*CC*	CC	AA	CC	AA	CC
*12)*	*g.-839G>A(R)* ^a^	*g.363G>A*	*GG*	GG	AA	GG	AA	GG
*13)*	*g.-870T>G(K)* ^a^	*g.332T>G*	*TT*	TT	GG	TT	GG	TT
*14)*	*g.-890C>T(Y)* ^a^	*g.312C>T*	*CC*	CC	TT	CC	TT	CC
15)	*g.-924G* *≥* *A(R)*	*g.278G>A*	*GG*	GG	AA	GG	AA	GG
16)	*g.-929A* *≥* *T(W)*	*g.273A>T*	*AA*	AA	TT	AA	TT	AA
17)	*g.-931A* *≥* *C(M)*	*g.271A>C*	*AA*	AA	CC	AA	CC	AA
*18)*	*g.-950DelA* ^a^	*g.252DelA*	*AA*	AA	DelA	AA	DelA	AA
*19)*	*g.-956DelG* ^a^	*g.246DelG*	*GG*	GG	DelG	GG	DelG	GG
20)	*g.-968A* *≥* *C(M)*	*g.234A>C*	*AA*	AC	CC	AC	CC	*AC/CC*
*21)*	*g.-973G>A(R)* ^a^	*g.229G>A*	*GG*	GG	AA	GG	AA	GG
*22)*	*g.-974DelG* ^a^	*g.228DelG*	*GG*	GG	DelG	GG	DelG	GG
*23)*	*g.-975DelG* ^a^	*g.227DelG*	*GG*	GG	DelG	GG	DelG	GG
*24)*	*g.-976DelG* ^a^	*g.226DelG*	*GG*	GG	DelG	GG	DelG	GG
*25)*	*g.-977G>T(K)* ^a^	*g.225G>T*	*GG*	GG	TT	GG	TT	GG
*26)*	*g.-978G>T(K)* ^a^	*g.224G>T*	*TT*	TT	GG	TT	GG	TT
27)	*g.-981C* *≥* *G(S)*	*g.221C>G*	*CC*	CC	GG	CC	GG	*CG*
28)	*g.-982T* *≥* *G(K)*	*g.220T>G*	*TT*	TT	GG	TT	GG	*TG*
29)	*g.-983T* *≥* *C(Y)*	*g.219T>C*	*TT*	TT	CC	TT	CC	*CT*
30)	*g.-984T* *≥* *A(W)*	*g.218T>A*	*TT*	TT	AA	TT	AA	*AT*
31)	*g.-985G* *≥* *T(K)*	*g.217G>T*	*GG*	GG	TT	GG	TT	*GT*
32)	*g.-986T* *≥* *C(Y)*	*g.216T>C*	*TT*	TT	CC	TT	CC	*AT*
33)	*g.-990C* *≥* *G(S)*	*g.212C>G*	*CC*	CC	GG	CC	GG	*CG*
34)	*g.-1024T* *≥* *C(Y)*	*g.178T>C*	*TT*	CT	CC	CT	CC	*CT*
35)	*g.-1029A* *≥* *G(R)*	*g.173A>G*	*AA*	AG	GG	AG	GG	*AG*
*36)*	*g.-1042C>A(M)* ^a^	*g.160C>A*	*CC*	CC	AA	CC	AA	CC
*37)*	*g.-1060C>T(Y)* ^a^	*g.142C>T*	*CC*	CC	TT	CC	TT	CC
*38)*	*g.-1070G>A(R)* ^a^	*g.132G>A*	*GG*	GG	AA	GG	AA	GG
*39)*	*g.-1077C>T(Y)* ^a^	*g.125C>T*	*CC*	TT	–/–	TT	–/–	CC
*40)*	*g.-1093G>A(R)* ^a^	*g.109G>A*	*GG*	AA	–/–	AA	–/–	GG
*41)*	*g.-1095G>T(K)* ^a^	*g.107G>T*	*TT*	GG	–/–	GG	–/–	TT
42)	*g.-1096C>A(M)* ^a^	*g.106C>A*	CC	AA	–/–	AA	–/–	CC

SNPs occur in Duroc and crossbreed pigs are underlined

^a^SNPs not detected in crossbreed pigs are italicized

^b^Numbering of SNPs submitted in dbSNP/NCBI database

^c^Numbering according to promoter sequence U39198, GenBank (Szafranska et al. 2001)

^d^BAC clone (CH242-294016) containing *pPAG3* gene sequences

^e^BAC clone (CH242-60C13) containing *pPAG3* and *pPAG6* gene sequences

Surprisingly, 42 SNPs with the Duroc gDNA template were discovered, including 24 novel SNPs (Table 2), not identified in crossbreed pigs. It was also found that 18 SNPs occurred in both Duroc and crossbreed pigs (underlined in Table 2). Among 42 SNPs, five deletions, *g.-950DelA*; *g.-956DelG*; *g.-974DelG*; *g.-975 DelG*, and *g.-976DelG*, were identified, 17 TRNs and 19 TRVs and one insertion (*g.-101_-102InsG*), which was also discovered in all crossbreed pigs (Table 2). Further evidence of the *pPAG2-L* genetic diversity provided a comparative analysis of the SNPs, which were identified in the promoter sequences of BAC clones (Duroc) and crossbreed pigs (Table 2).

### *In silico* identification of various TFBSs in the *pPAG2-L* promoter sequence of the crossbreeds

**Fig. 3:**
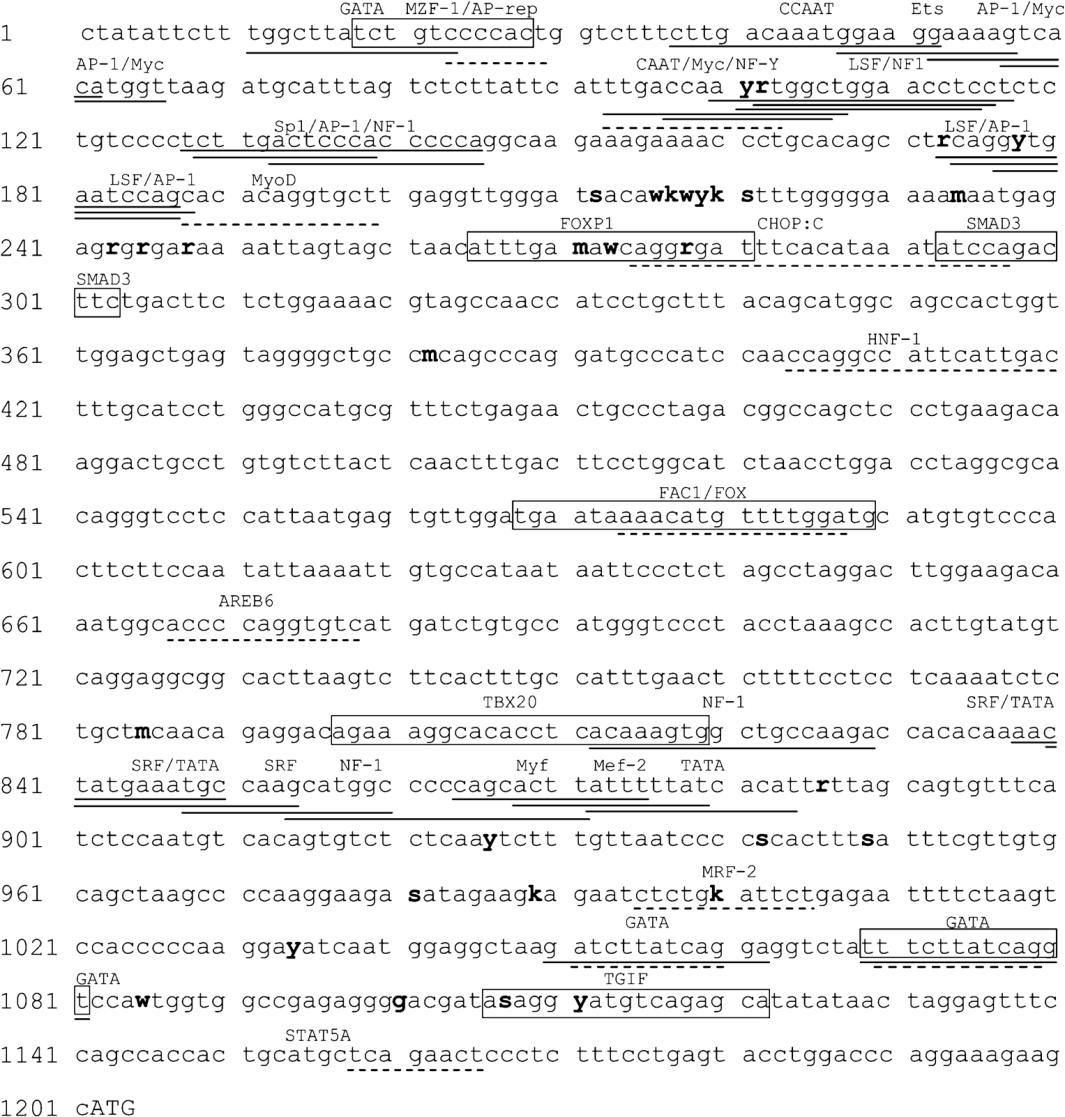
Promoter sequence (1204 bp with ATG) of the *pPAG2* gene (U39198; GenBank) containing identified SNPs (acc. IUPAC code; *bolded line*) and TFBS predicted with the use of Lasagne-Search 2.0 (*dashed line*), Cluster Buster (*solid line*), and MatInspector (*boxed*)

The investigations for the vertebrate-specific promoter elements by Lasagne-Search 2.0 (using 259 motifs from TRANSFAC^®^ application) in the *pPAG2-L* promoter sequence revealed 11 various TFBSs (Table 3, Fig. 3). Among of all identified motifs, only two were identified on the sense strand, whereas nine were on anti-sense strand (*p* value = 0, *e* value = 0, and score from 8.14 to 18.64). It was confirmed that allel G (*g.-221G>C*; according to IUPAC numbering in Table 1) created the GATA core motif, while the C allel was unable to create such TFBS (Fig. 1). Three SNPs, *g.-1101T>C(Y)*, *g.-924G>A(R)*, and *g.-202T>G(K)*, appeared in the TFBS core sequences of NF-Y, CHOP:C, and MRF-2, respectively. The other SNPs occurred within AP-2rep, MyoD, HNF-1, FAC1, and AREB6, but STAT5A occurred outside of the TFBS core sequences.

**Table 3:** Prediction of transcription factors (TF) binding sites with the context of novel SNPs [in brackets] identified *in silico* in the promoter of the *pPAG2-L* gene with the use of Lasagne-Search 2.0, Cluster Buster, and MatInspector

TF motif	Localization	Strand	Score	TF sequence
Lasagne-Search 2.0				
AP-2rep	23–29	−	8.14	**CAGTGGG** ^b^
NF-Y	93–103	+	13.05	**TGACCAA[T/C]**[G/A]TG
MoyD	188–199	+	11.99	ACA**CAGGTG**CTT
CHOP:C/EBPalpha	275–297	−	13.2	ATG**TGAAATC[C/T]** *CC*
HNF-1	404–420	−	14.2	A**GTCAAT**GAATGGCCTG
FAC1	574–587	−	10.21	ATCC**AAAACATGTT**
AREB6	667–678	−	11.89	TGA**CACCT**GGGG
MRF-2	995–1005	−	18.64	CAG**AAT[A/C]C**AGA
GATA-2	1051–1060	−	11.32	CCT**GATAAGA**
GATA-2	1070–1079	−	11.32	CCT**GATA**AGA
STAT5A	1158–1165	−	8.85	GAG**TTCT**G
Cluster Buster				
GATA	11–23	−	6.87	GACA**GATA**AGCCA
CCAAT	37–52	+	6.16	CTTGA**CAAAT**GGAAGG
Ets	47–57	+	8.18	GGAA**GGAA**AAG
AP-1	52–62	−	4.49	TG**TGAC**TTTTC
Myc	57–66	−	7.12	AC**CATGTG**AC
CCAAT ^a^	92–107	+	4.28	TTTGA**CCAA[T/C]**[G/A]TGGCT
Myc	97–106	+	4.58	CC**AA[T/C][G/A]TG**GC
LSF	101–115	+	5.26	[T/C][A/G]TGGCTGGAACCTC
LSF	102–116	−	5.85	GGAGGTTCCAGCCA[T/C]
NF-1	128–145	+	4.65	**TCTTGAC**TCCCACCCCCA
AP-1	129–139	+	4.63	CT**TGA** *C*TCCCA
Sp1	133–145	−	7.06	TGG**GGGTGGG**AGT
LSF	173–187	+	5.45	[A/G]CAGG[T/C]TGAATCCAG
LSF	174–188	−	8.86	GCTGGATTCAACCTG
**NF1** ^c^	100–117	+	5.25	A[C/T][**A/G]TGGC**TGGAACCTCCT
**AP-1**	177–187	+	4.12	GC**[T/C]GAA**TCCAG
NF-1	812–829	−	7.85	TC**TTGG**CAGCCACTTTGT
SRF	838–850	+	5.33	AA**CTATGAAATG**C
TATA	840–854	+	4.45	C**TATG**AAATGCCAAG
SRF	848–860	+	5.9	TG**CCAAGCATGG**C
NF-1	854–871	+	5.03	GC**ATGGC**CCCCAGCACTT
Myf	864–875	−	4.46	AAATAAGTGCTG
Mef-2	868–879	+	7.84	AC**TTATTTTTAT**
GATA	872–884	−	6.1	ATGT***GATA***AAAAT
GATA	1050–1062	−	6.94	TCCT***GATA***AGATC
GATA	1069–1081	−	8.43	ACCT***GATA***AGAAA
MatInspector				
MZF1.01	18–28	−	1.0	GT**GGGG**ACAGA
FOXP1 ES.01 ^a^	265–281	+	1.0	ATTTGA[A/C]**A[A/T]CA**GG[G/A]GAT
SMAD3.01	293–303	−	1.0	GAA**GTCT**GGAT
FOXP1 ES.01	568–584	+	1.0	TGAATA**AAACA**TGTTTT
FOXP1 ES.01	573–589	−	1.0	CATCCA**AAACA**TGTTTT
TBX20.01	797–819	−	1.0	CACTTTGTG**AGGT**GTGCCTTTCT
GATA.01	1069–1081	−	1.0	ACCT**GATA**AGAAA
*TGIF.01* ^d^	1106–1122	+	1.0	A[C/G]AGG[C/T]A**TGTCA**GAGCA

^a^Underlined motifs are the TFBS with SNPs in the core sequence

^b^Bolded letters are the core nucleotides

^c^Bolded and underlined motifs are the new TFBS that appeared as a result of SNP occurrence

^d^Underlined and italic motifs are the TFBS with SNP outside the core sequence

The Cluster Buster revealed 12 TFBS in three clusters (C1–C3) including 11–188 bp (C1), 1050–1081 bp (C2), and 812–884 bp (C3) of the *pPAG2* promoter (Table 3). The score of motifs was 4.12–8.18 for sense strand and 4.46–8.86 for the anti-sense strand. Some of TFBS were identified in various regions, e.g., GATA (C1: 11–23; C2: 1050–1062 and 1069–1081; C3: 872–884) or NF-1 (C1: 100–117, 128–145, C3: 812–829 and 854–871). Only two SNPs, *g.-1100G>A (R)* and *g.-1101 T>C (Y)*, were identified within NF1 and AP-1 motifs.

The MatInspector/Genomatix confirmed eight major TFBSs (Table 3). The SNP (*g.-929A>T(W)/g.273A>T*) was identified within core AACA (272–275) of the Fork head domain factors (FOXP1; 265–281), whereas two SNPs, *g.-931A>C(M)* and *g.-924G>A(R)*, were outside the core sequence. Furthermore, two SNPs *g.-95C>G(S)* and *g.-91C>T (Y)* were identified outside the GTCA core sequence (1114–1117) of the TALE homeodomain class recognizing TG motifs (TGIF). There was no prevalence of SNPs in the sequences coding other TFBSs: MZF1 (18–28), SMAD3 (293–303), FOXP1 (573–589), TBX20 (797–819), and GATA (1069–1081).

## Discussion

In total, 31 SNPs and one InDel (Table 1, Fig. 1) were identified in the *pPAG2-L* promoter (up to −1100 upstream ATG), including 19 SNPs in the F2 (15 SNPs in three clusters, and another four SNPs occurring in tandems). In F1, 13 SNPs were identified, including one InDel (*g.-101/102InsG*) inside the GATA sequence (*p*_*x*_ = 0.86).

Currently, the 32 polymorphisms of the *pPAG2-L* promoter (from *g.-91C>T(Y)* to *g.-1101T>C(Y)* identified in crossbreed pigs can only be compared to the five deposited promoter sequences: *pPAG2* (Szafranska et al. 2001), *bPAG1*, *ePAG*, *bPAG2* (Xie et al. 1995; Green et al. 1999; Telugu et al. 2009), and *fPAG* (Ensembl). However, a comparison of the SNPs in the *pPAG2-L* promoter sequence is impossible, because the SNPs of *bPAG1*, *ePAG*, and *bPAG2* have not been studied.

Surprisingly, a comparative analysis of identified BAC clone sequences revealed 97.0–99.3 % homology to the *pPAG2* promoter (Szafranska et al. 2001), which suggests SNPs among different breeds. The sequence diversity of two BAC clones originating from Duroc (used as gDNA control templates) revealed 42 polymorphisms (36 SNPs and 6 InDel; Table 2), among which novel 24 SNPs have also been identified in the crossbreed pigs (Table 1). It should be noted that some identified SNPs in crossbreed pigs were identical as in the BAC clones (Duroc), which undoubtedly indicates that the currently-tested crossbreed pigs were interbreeding hybrids between Duroc with other breeds. Thus, the identified SNPs are evidence of duplication and positive selection of the *pPAG2-L* subfamily in different breeds.

Previously, specific sequences of the various TFBSs were identified in porcine *pPAG2*: Ets, Ets-1, GATA, GATA-like, and STAT (Szafranska et al. 2001). Presently, the location of SNPs identified in the *pPAG2-L* promoter regulatory F1 region suggests importance due to the potential impact on the TFBSs and, consequently, transcription activation. *In silico* analysis using three programs (Table 3) revealed a total of 28 various TFBSs. In the F1, we found conserved sequences for TATATAA box (from −73 to −79 bp), AP1 (activator protein 1) and CCAAT (enhancer binding protein (C/EBP). The Lasagne 2.0 software was able to detect SNPs (*g.-1101T>C*, *g.-924G>A*, and *g.-202T>G*), which diminish binding sites for NF-Y, CHOP:C, and MRF-2, which may have an influence on the *PAG2-L* expression in these three heterozygote genotypes: CT (*p*_*x*_ = 0.12), AG (*p*_*x*_ = 0.29), and TG (*p*_*x*_ = 0.44), respectively. Cluster Buster also confirmed that two SNPs, *g.-1000G>A(R)* and *g.-1101T>C(Y*), may affect the *PAG2-Ls* expression by NF1 and AP-1.

Furthermore, the presence of AP-2 transcription factor was detected, which was also found in the promoters of some placental bovine genes, especially *bPAG1* and *bPAG17*. This suggests that the AP-2 family is a major factor regulating genes depending on cytochrome P-450 involved in the production of steroid hormones in the binucleated cells (Ushizawa et al. 2007). The significant evidence of the *PAG* family involvement in the regulation of pregnancy maintenance has provided commercial bovine microarrays containing 1780 genes, including 30 expressed genes (25–250 dpc), mainly in the bovine two-nucleated placental cells (Ushizawa et al. 2007). Moreover, *Affymetrix* microarrays showed a significant correlation of the *bPAG11* with prostaglandins (PG) synthesis: PGE synthase (*r* = 0.76), cytosolic PGE synthase 3 (*r* = 0.69), and endoperoxide synthase 2 (*r* = 0.86), suggesting an important role of the *bPAG11* in the PG cascade activation (Thompson et al. 2011). It is necessary to underline that two presently identified SNPs in the *pPAG2-L* promoter (*g.-117A>T* and *g.-168C>T*) were localized within, or almost nearby, 10 nt unique tandem repeats (TCTTATCAGG located at - 94 to - 103 and - 113 to - 122 upstream of ATG), which are specific in the activation of the PAG gene family in pigs (Szafranska et al. 2001) or/and cattle (Telugu et al. 2009), respectively. Both of these SNPs identified in crossbreed pigs are very close to the conservative Ets sequence with proximity to the GATA within the pPAG2 promoter recognized previously (Szafranska et al. 2001). In cattle, the Ets analyzed in eight known bPAG promoters maintained conservative sequences (Telugu et al. 2009). Thus, these SNPs may affect placental development and pregnancy maintenance in both species.

Although there was no prevalence of SNPs in the GATA sequence of the *pPAG2-L* promoter, the Cluster Bluster revealed that allele A (SNP *g.-1100G>A)* creates NF1, while allele G (genotype GG) does not create this TFBS. However, the frequency of heterozygote genotype GA and TC was, in both cases, *p*_*x*_ = 0.12; the frequency of homozygote genotypes GG and TT (which do not determine TFBS) was *p*_*x*_ = 0.88. The SNPs *g.-1100G>A(R)* that appeared inside the core determined the occurrence of a new TFBS (NF-1).

In contrast, the MatInspector revealed sequences characteristic for three variously located *FOXP1 ES.01*, although only one SNP (*g.-929A>T(W)/g. 273A>T*) was localized in the core AACA sequence of this TFBS (265–281 bp). Previous studies have shown that *FOXP1* deletion has an embryonic lethal defect that affects a variety of organs, including cardiac (Wang et al. 2004) and lung development (Shu et al. 2007), and B cell differentiation (Hu et al. 2006). The SNPs within the *pPAG2-Ls* promoter (from *g.-990C>G* to *g.-954A>G*; *g.-820C>A*; *g.-417C>A*; *g.-276T>C* to *g.-213T>G*; *g.-168C>T*; and *g.-117A>T* to *g.-91C>T*) did not influence or affect the appearance of TFBSs. The other TFBSs identified *in silico* is Tbx20 – a transcription factor that is essential for proper heart development in a growing fetus (Song et al. 2006).

The participation of many transcription factors involved in *bPAG* activation has been shown by EMSA (electrophoretical mobility shift assay), and the most important was assigned to the Ets2 and DDVL – drosophila dorsal ventral factor (Telugu et al. 2009), as well as in the regulation of transcription of chorionic genes, e.g., IFNτ in cattle (Ezashi et al. 1998) or hCG in women (Ghosh et al. 2003). Presently, in crossbreed pigs, no SNPs were identified in the Ets2 binding site, although the identified SNPs in the GATA suggest the possibility of disturbance during *pPAG2-L* subfamily activation. In addition, the bovine microarrays (Kumar et al. 2010) indicate a strong influence of the STAT Pax-2 (signal transducer and activator of transcription; paired homeobox 2) on the promoters of genes that are expressed in the placenta, e.g., *bPAG2*, *PTGS2* (*COX2*, *PG –* *endoperoxide synthase 2*) and *LSG 34F*, as a homologue of a secretory vesicle protein in the male (SSLP-1; *seminal vesicle protein secreted*).

The investigation of SNP spreading in the selected population requires an important parameter – MAF (minor allele frequency) at the level of >0.1 in commercial pig breeds (McLaren et al. 2013). Although in our study, the MAF was not specified, among 32 SNPs, we are able to identify the genotype frequency *p*_*x*_ = 0.56–0.88 for 18 SNPs, which indicates the dominance of homozygotes, while in the case of 14 SNPs it indicates the dominance of heterozygotes (*p*_*x*_ = 0.55–1).

It is well known that the smallest polymorphism results from homozygosity of different domestic pig breeds. The occurrence of 1.2 SNPs/kbp in the genome of European Large White breed and only 0.05 SNPs/kbp in the genome of *Sus barbatus* indicates the inbreeding of pigs from the isolated populations, as a result of natural barriers or a human economic activity (Ferreira et al. 2008). The regions of homozygosity (ROHs) in European, Asian, and wild pigs (60 K SNP microarray) vary about 778.8 ± 349 ROHs/genome (one ROHs range between 10 kbp to 83.6 Mbp; average 1.11 Mbp), which represents about 23 % of the porcine genome (Bosse et al. 2012). A higher level of ROHs occurs in genomes of wild and domestic European pigs, while the lowest ROHs level (and the largest polymorphism) is present in the genome of wild Asian pigs. Furthermore, 1733 SNP ± 0.57/kbp occur in the porcine genome, but only 2.49 SNP ± 0.57/bp are in regions outside the ROHs (Bosse et al. 2012). This may suggest that the greatest heterozygosity of the *pPAG2-L* promoter occurs within various areas potentially located outside the ROHs in the crossbreed pigs.

## Conclusion

This study provides pioneering information on polymorphism and hints at the discovery of 32 SNPs/InDel identified within the regulatory proximal and flanking distal regions of the *pPAG2-L* promoter subfamily in crossbreed pigs and 42 SNPs/InDels identified in the Duroc breed (as inserts in BAC clones used as controls). Many of the *pPAG2-L* SNPs were identified in various TFBSs (at least 8 to 26, due to the three softwares used), which suggests the high importance of allelic (homo- and heterozygotic) diversity and meaningful influence on transcriptional regulation of the *pPAG2-L* subfamily expression.

Since this is the first study describing the *pPAG2-L* subfamily diversity in the genome of crossbreed pigs, it therefore also increased/extended the general knowledge on the last version of the domestic pig genome (Ss10.2). The results present a broad-based novel database (main pattern) – as the widest genotyping prototype, which is required for further investigations of various potential correlations between guiding SNP genotypes of the *pPAG2-L* subfamily in the sows of many breeds, in which the most economically important reproductive traits are properly documented on each farm of female and male progenitors.
